# Supplementary material for: A quaternary tetramer assembly inhibits the deubiquitinating activity of USP25
Source: Nat Commun. 2018 Nov 26;9:4973. doi: 10.1038/s41467-018-07510-5 (PMC6255862; doi:10.1038/s41467-018-07510-5)
Supplement: Supplementary file 1 — Supplementary Information [file 41467_2018_7510_MOESM1_ESM.pdf]

SUPPLEMENTARY INFORMATION

A quaternary tetramer assembly inhibits the deubiquitinating activity of USP25

Liu B. et al.

a

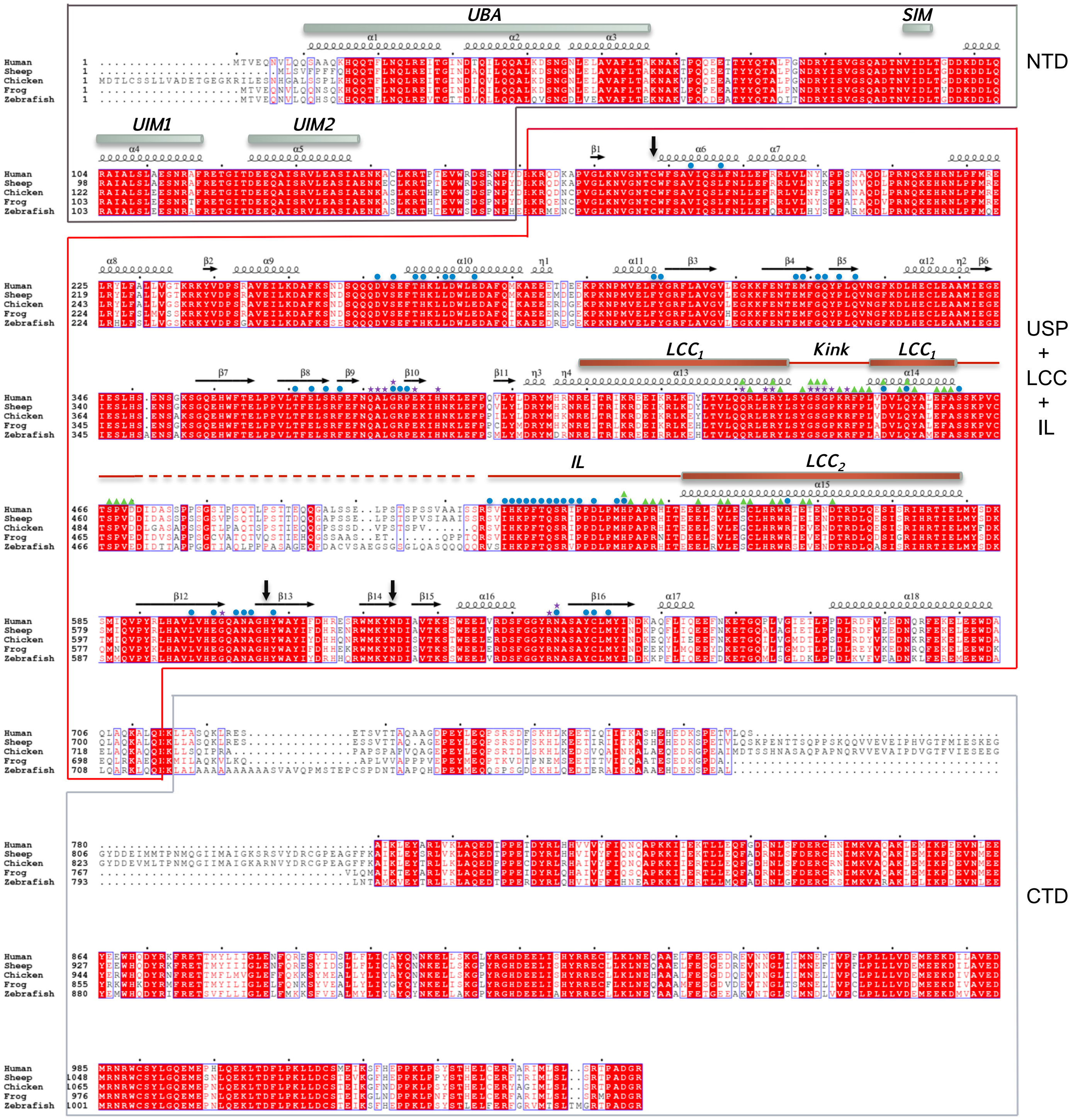

UBA
SIM
UIM1
UIM2
NTD
USP
+
LCC
+
IL
LCC1
Kink
LCC1
IL
LCC2
CTD
Human
Sheep
Chicken
Frog
Zebrafish

b

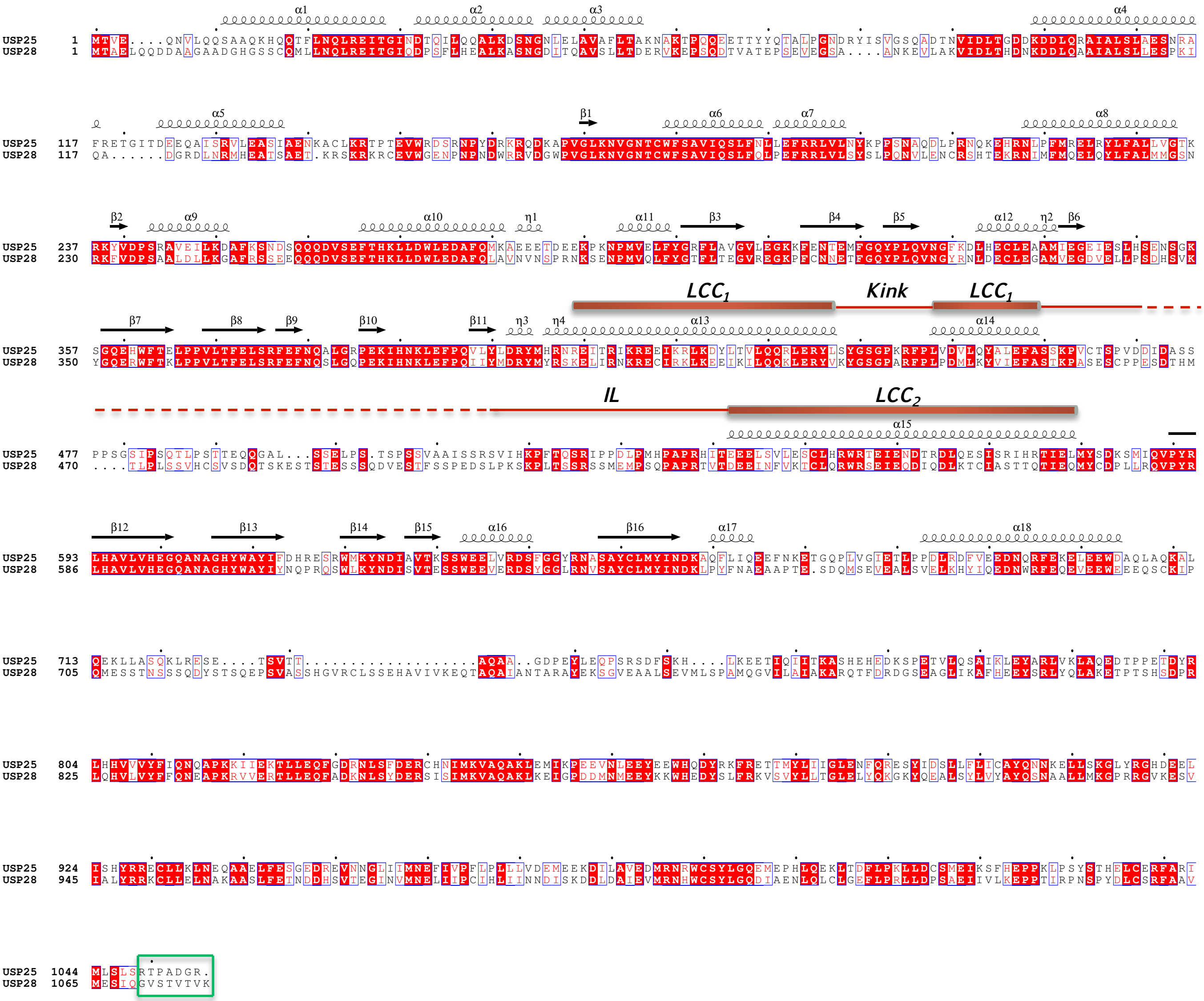

C

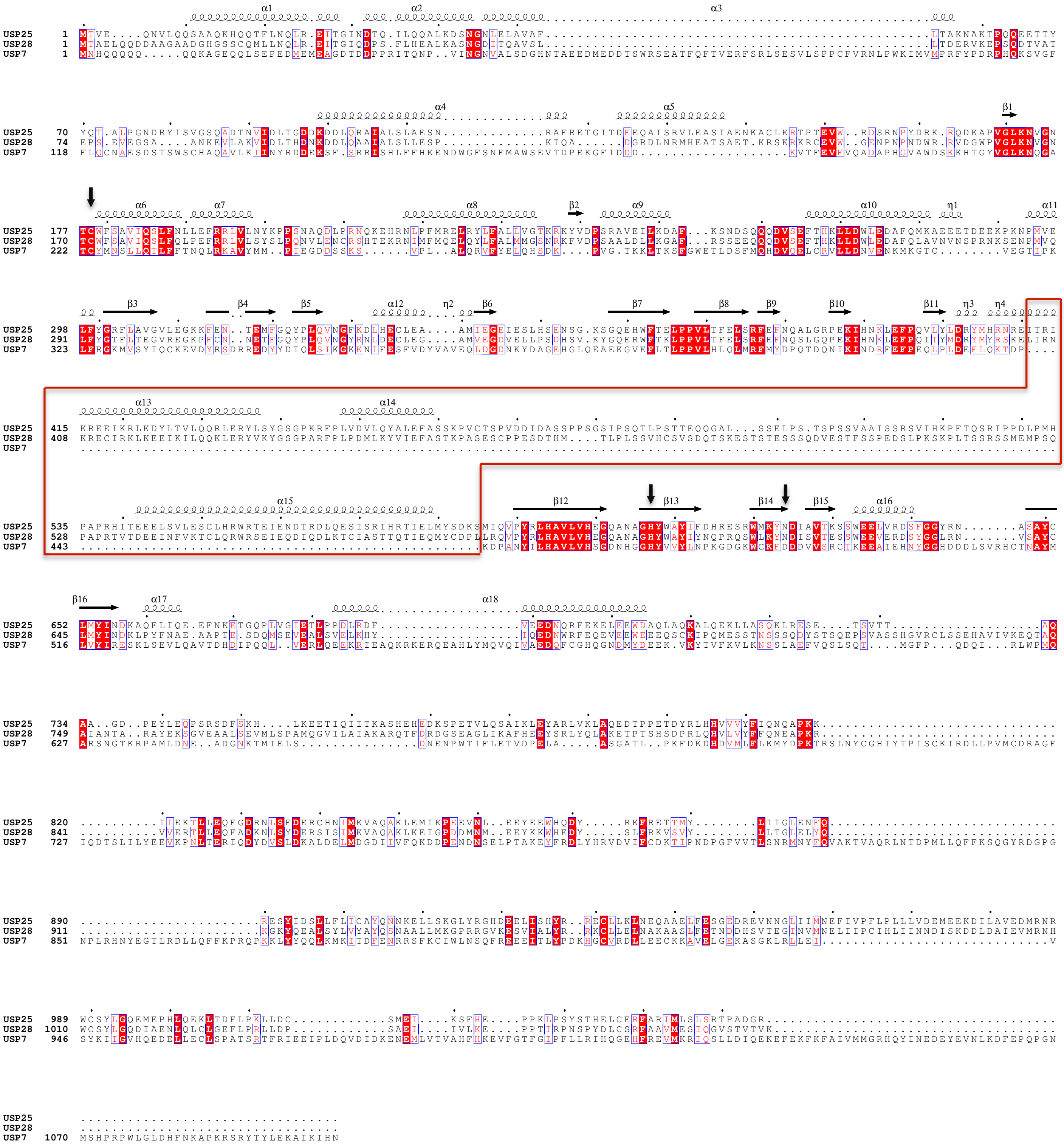

USP25
USP28
USP7
α1 α2 α3 α4 α5 β1
α6 α7 α8 β2 α9 α10 η1 α11
β3 β4 β5 α12 η2 β6 β7 β8 β9 β10 β11 η3 η4
α13 α14
α15 β12 β13 β14 β15 α16
β16 α17 α18

Supplementary Figure 1. Sequence alignments of USP25.

(a) Sequence alignment of human USP25 with its homologs in sheep, chicken, frog and zebrafish. Conserved residues are shaded in red. Three different domains of USP25 are labeled: N-terminal domain (labeled as NTD), USP-like domain (labeled as USP+LCC+IL) and C-terminal domain (labeled as CTD). Motifs in NTD are indicated in grey cylinders: ubiquitin associated domain (UBA), SUMO interacting motif (SIM) and two ubiquitin interacting motifs (UIM1 and UIM2). Long coiled coils (LCC_1 and LCC_2) are indicated in red cylinders, whereas the “Kink” and inhibitory loop (IL) are indicated in red lines. Dashed red line indicates the non-conserved sequence (absent in the USP25 crystal structure) of IL. Catalytic triad residues (C178, H607 and N624) are highlighted with black arrows. Residues involved in the interactions between molecules B and A (tetramer contacts IL-loop), molecules B and A’ (tetramer contacts Kink), and molecules B and B’ (dimer contacts) are marked with blue dots, purple stars and green triangles, respectively. Molecules A, A’, B and B’ are indicated in **Fig. 3b** and **Fig. S5**. (b) Sequence alignment of USP25 with its homolog USP28. The labeling schemes for LCC_1, LCC_2, “Kink” and IL are the same as in (a). The C-terminal tails of USP25 (residues interacting with tankyrases) and USP28 are marked with a green square. (c) Sequence alignment of USP25 with USP28 and USP7. Catalytic triad residues are highlighted with black arrows. The long sequence insertions of USP25 and USP28 are circled with red lines. All sequences are aligned online with Clustal Omega and formatted using ESPript.

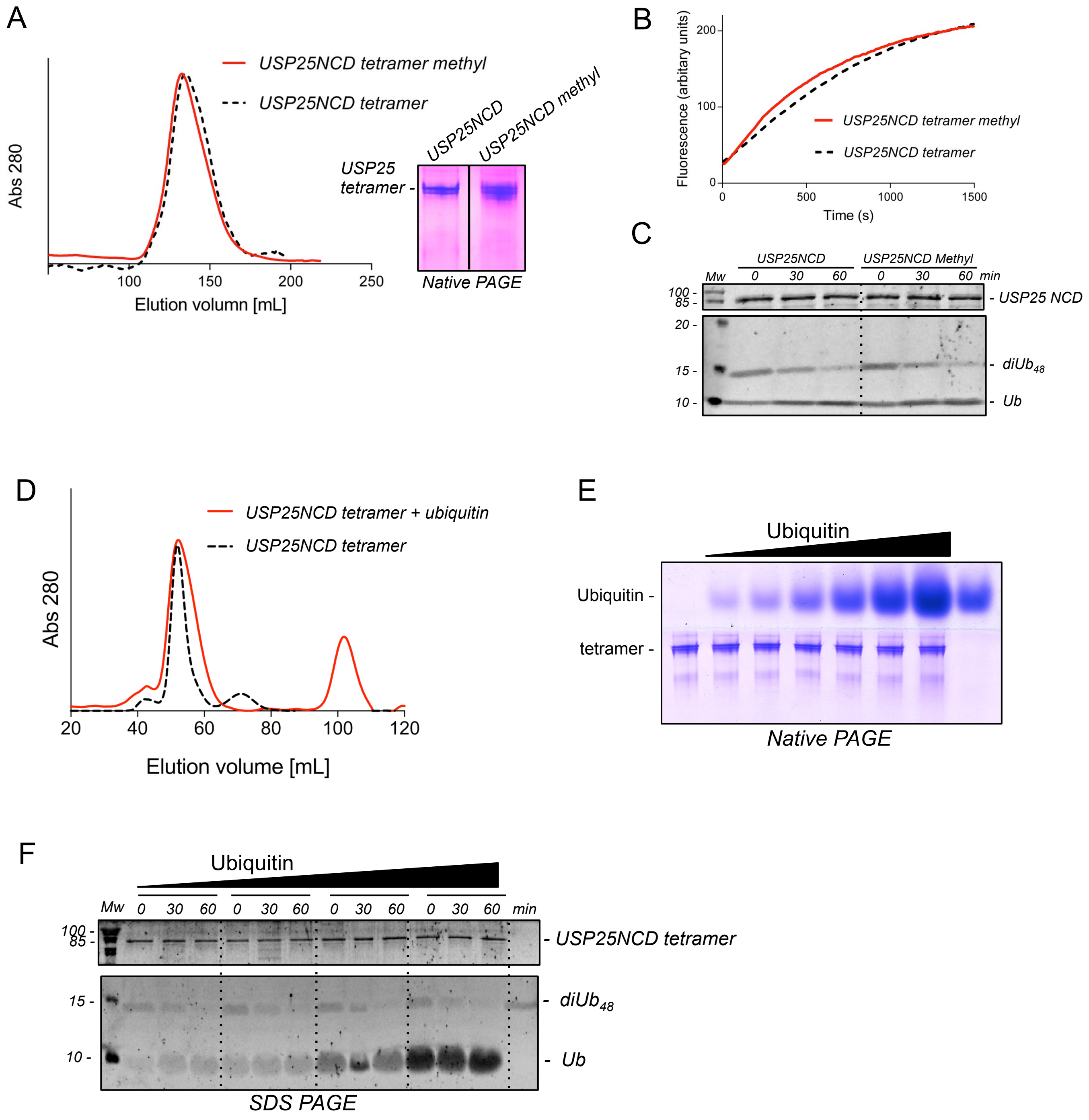

Supplementary Figure 2. Biochemical characterization of methylated USP25 and competition with ubiquitin.

(A) Gel filtration profiles of the USP25NCD tetramer before and after conducting the lysine methylation protocol described in the methods. ***Right***, native PAGE of USP25NCD tetramer before and after conducting the lysine methylation protocol. (B) Time-course deubiquitinating activity of the USP25NCD, before and after conducting the lysine methylation protocol, using Ub-AMC fluorescent substrate. (C) PAGE analysis of a time-course deubiquitinating activity of the USP25NCD, before and after conducting the lysine methylation protocol, using $diUb_{48}$ as a substrate. (D) Gel filtration profiles of the USP25NCD in the presence of ubiquitin. (E) Native gel of the USP25NCD tetramer in the presence of increasing amounts of ubiquitin. (F) PAGE analysis of a time-course deubiquitinating activity of the USP25NCD, in the presence of increasing amounts of ubiquitin, using $diUb_{48}$ as a substrate.

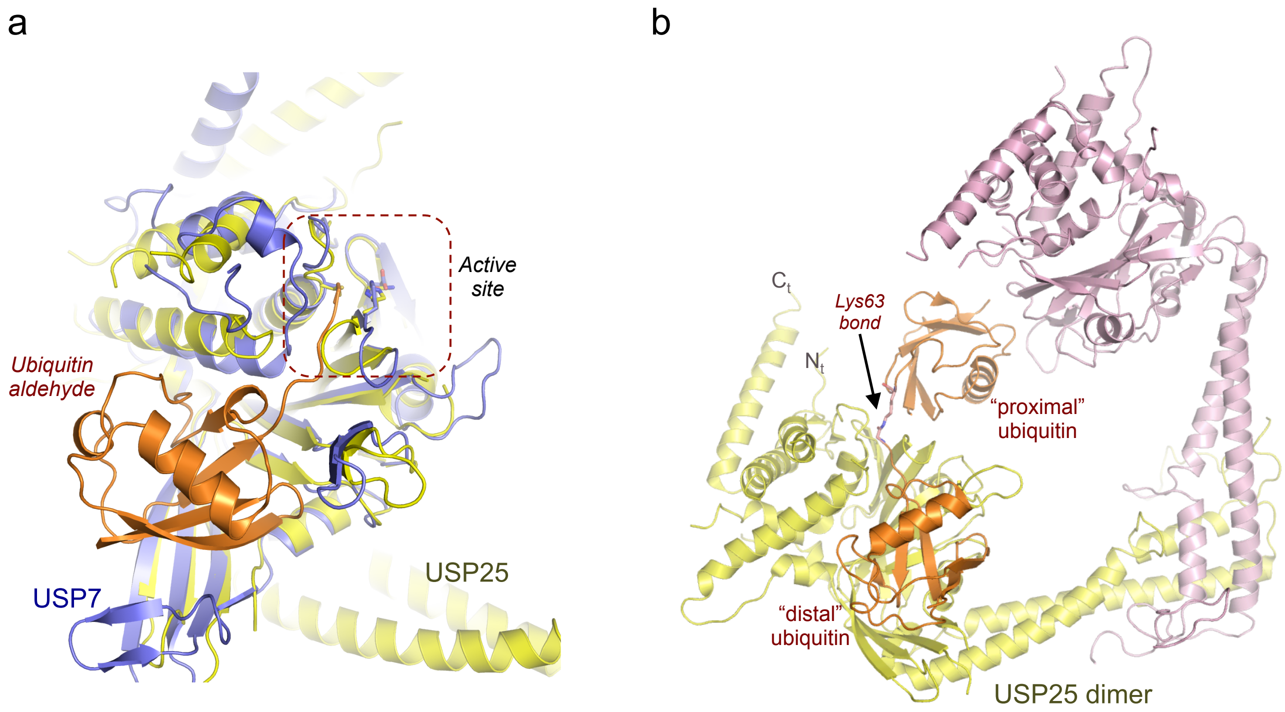

Supplementary Figure 3. Structure comparison of USP25 with USP7-ubiquitin aldehyde and CYLD-K63 diubiquitin complexes.
(a) Superimposition of the catalytic region of USP25 and USP7-ubiquitin aldehyde complex. USP25 is shown in yellow. USP7-ubiquitin aldehyde complex (PDB code 5JTJ) are shown in blue and orange for each molecule. Active site catalytic triad residues are shown in stick representation. Zoomed-up details of the active site region depicted in **Fig. 2d**. (b) Structural model of K_{63}-diubiquitin substrate in complex with USP25 dimer, based on the superposition between USP25 and the CYLD-K63-diubiquitin structure (PDB code 3WXG). Rmsd of the superposition is 2,74 Å for 218 aligned residues. "Proximal" and "distal" ubiquitin are shown in orange.

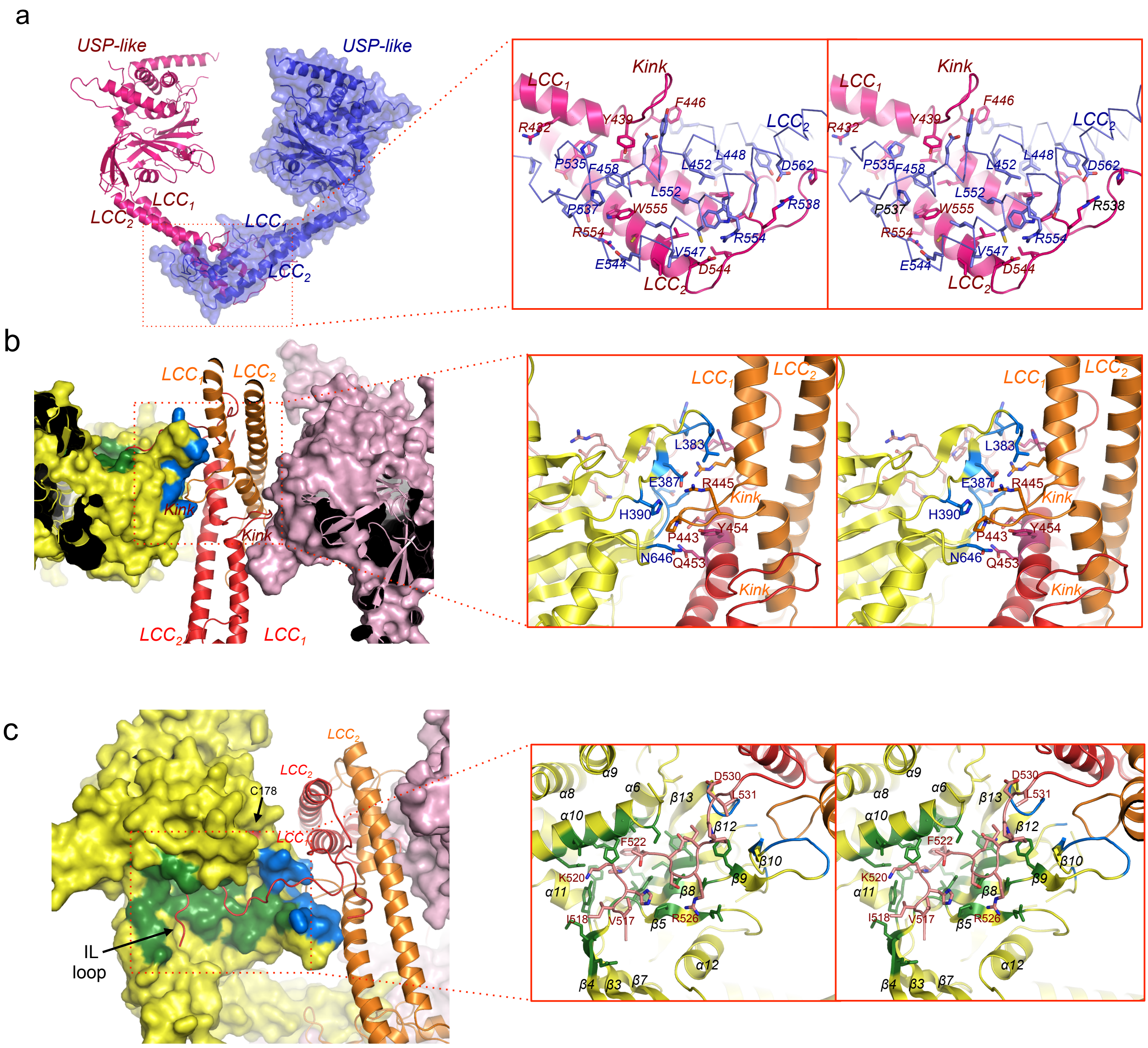

Supplementary Figure 4. Detailed interactions of the residues composing the interfaces in the dimers and tetramer assemblies of USP25.
(a) *Left*, ribbon and surface representation of the two monomers composing the dimer structure observed in the crystal structure of USP25. *Right*, stereo representation of the interface between the LCC domains in the dimer assembly. Contact residues are labeled and shown in stick representation. (b) *Left*, overall structure of the "*kink*" interaction with the USP25 catalytic domain. The interaction surface is shown in blue. *Right*, stereo representation of the details of interaction. Contact residues are depicted in stick representation. "*Kink*" residues are labeled in orange, whereas the interaction residues in catalytic domain are labeled in blue. (c) *Left*, overall structure of IL-Loop interaction with the USP25 catalytic domain. The interaction surface is shown in green. *Right*, stereo representation of the details of interaction. Contact residues are depicted in stick representation. IL-loop residues are labeled in orange, whereas the interaction residues in catalytic domain are labeled in green.

a

Tetramer interaction (IL-loop)

Chain B			Chain A			Distance(Å)
VAL	183	CG2	PHE	522	CG	3.13
LEU	187	CD2	PHE	522	CE2	3.68
ASP	262	OD2	ILE	527	CG2	3.32
SER	264	OG	GLN	524	N	2.68
SER	264	OG	THR	523	CB	3.31
THR	267	OG1	THR	523	CG2	3.4
HIS	268	NE2	LYS	520	CG	3.27
HIS	268	CA	THR	523	CG2	3.81
LEU	271	CD1	LYS	520	CB	3.49
LEU	271	CD1	PRO	521	N	3.71
LEU	271	CD1	PHE	522	N	3.78
ASP	272	OD1	LYS	520	CE	3.08
GLU	275	OE2	LYS	520	CE	3.21
PHE	299	CG	PRO	521	CG	3.98
TYR	300	OH	LYS	520	CD	2.91
TYR	300	CE2	ILE	518	CG2	3.47
GLU	318	OE1	SER	516	CB	3.17
MET	319	O	ILE	518	CG1	3.63
GLY	321	N	ILE	518	CG2	3.97
GLY	321	CA	HIS	519	O	3.2
GLN	322	NE2	THR	523	O	2.64
GLN	322	N	HIS	519	O	2.99
GLN	322	OE1	PRO	521	O	2.32
GLN	322	CD	LYS	520	O	3.55
PRO	324	CG	HIS	519	CD2	3.2
PRO	324	CB	ARG	526	NH2	3.76
GLN	326	CG	ARG	526	NH1	3.13
THR	371	OG1	PRO	521	O	3.86
GLU	373	OE2	GLN	524	CA	3.62
GLU	373	OE1	SER	525	OG	2.68
GLU	373	CG	THR	523	O	3.71
SER	375	OG	ARG	526	CB	3.42
SER	375	OG	SER	525	O	3.93
PHE	377	CB	ARG	526	O	3.67
PHE	377	CD1	PRO	528	CD	3.66
GLY	384	CA	HIS	534	NE2	3.14
ARG	385	CG	MET	533	SD	3.58
PRO	386	CG	PRO	528	CB	3.85
ASP	450	OD1	ASN	646	ND2	3.49
GLN	453	NE2	GLU	600	CG	3.65
GLN	453	OE1	ASN	646	ND2	2.72
SER	460	OG	ALA	605	CB	3.94
SER	516	CB	GLU	318	OE1	3.17
ILE	518	CG2	TYR	300	CE2	3.47
ILE	518	CG2	GLY	321	N	3.97
ILE	518	CG1	MET	319	O	3.63
HIS	519	O	GLY	321	CA	3.2
HIS	519	CD2	PRO	324	CG	3.2
HIS	519	O	GLN	322	N	2.99
LYS	520	CE	GLU	275	OE2	3.21
LYS	520	CD	TYR	300	OH	2.91
LYS	520	CG	HIS	268	NE2	3.27
LYS	520	CE	ASP	272	OD1	3.08
LYS	520	O	GLN	322	CD	3.55
LYS	520	CB	LEU	271	CD1	3.49
PRO	521	N	LEU	271	CD1	3.71
PRO	521	O	THR	371	OG1	3.86
PRO	521	O	CYS	651	SG	3.77
PRO	521	O	GLN	322	OE1	2.32
PRO	521	CG	PHE	299	CG	3.98
PHE	522	CZ	MET	653	SD	3.65
PHE	522	CE2	LEU	187	CD2	3.68
PHE	522	O	TYR	650	CE2	3.74
PHE	522	N	LEU	271	CD1	3.78
PHE	522	CG	VAL	183	CG2	3.13
PHE	522	CD1	LEU	597	CD1	3.4
PHE	522	CD1	CYS	651	SG	3.75
THR	523	CG2	HIS	268	CA	3.81
THR	523	O	GLU	373	CG	3.71
THR	523	CG2	THR	267	OG1	3.4
THR	523	O	GLN	322	NE2	2.64
THR	523	CB	SER	264	OG	3.31
GLN	524	CA	GLU	373	OE2	3.62
GLN	524	N	SER	264	OG	2.68
GLN	524	OE1	TYR	608	OH	3.62
GLN	524	CG	TYR	650	CD2	3.76
SER	525	OG	GLU	373	OE1	2.68
SER	525	O	SER	375	OG	3.93
ARG	526	O	PHE	377	CB	3.67
ARG	526	NH1	GLN	326	CG	3.13
ARG	526	CB	SER	375	OG	3.42
ARG	526	NH2	PRO	324	CB	3.76

Chain B			Chain A			Distance(Å)
ILE	527	CG2	ASP	262	OD2	3.32
ILE	527	CD1	ALA	603	O	3.45
PRO	528	CB	PRO	386	CG	3.85
PRO	528	CD	PHE	377	CD1	3.66
ASP	530	OD2	ASN	604	ND2	2.69
MET	533	SD	ARG	385	CG	3.58
HIS	534	NE2	GLY	384	CA	3.14
ARG	556	NH2	GLU	600	OE1	2.8
LEU	597	CD1	PHE	522	CD1	3.4
GLU	600	CG	GLN	453	NE2	3.65
GLU	600	OE1	ARG	556	NH2	2.8
ALA	603	O	ILE	527	CD1	3.45
ASN	604	ND2	ASP	530	OD2	2.69
ALA	605	CB	SER	460	OG	3.94
TYR	608	OH	GLN	524	OE1	3.62
ASN	646	ND2	ASP	450	OD1	3.49
ASN	646	ND2	GLN	453	OE1	2.72
TYR	650	CD2	GLN	524	CG	3.76
TYR	650	CE2	PHE	522	O	3.74
CYS	651	SG	PHE	522	CD1	3.75
CYS	651	SG	PRO	521	O	3.77
MET	653	SD	PHE	522	CZ	3.65

Dimer interaction (LCC domain)

Chain B			Chain B'			Distance(Å)
GLN	431	O	HIS	534	NE2	3.93
ARG	432	NH2	PRO	468	C	3.26
ARG	432	NH1	ASP	470	CB	3.65
ARG	432	NH2	SER	467	C	3.14
ARG	432	NH2	VAL	469	N	3.42
ARG	432	CG	HIS	534	CE1	3.63
ARG	435	CG	PRO	535	CD	3.46
ARG	435	CB	HIS	534	CD2	3.19
TYR	436	OH	SER	467	CB	3.71
TYR	436	CE2	PRO	535	CB	3.6
TYR	436	OH	HIS	534	O	3.39
TYR	439	CE1	PRO	535	CG	3.89
TYR	439	OH	PHE	458	CA	3.23
TYR	439	OH	GLU	457	C	3.67
TYR	439	CE2	TYR	454	O	3.48
GLY	440	C	GLU	457	OE1	3.91
GLY	440	CA	TYR	454	CE2	3.39
SER	441	OG	GLN	453	NE2	3.6
SER	441	N	TYR	454	CE1	3.67
SER	441	OG	GLU	457	OE2	2.77
GLY	442	N	TYR	454	OH	3.11
LYS	444	O	TYR	454	OH	2.65
PHE	446	CE2	TYR	454	CE2	3.8
PHE	446	CZ	PRO	447	CD	3.82
PHE	446	CG	PHE	446	CG	3.69
PHE	446	CE2	ASP	450	CB	3.52
PRO	447	CD	PHE	446	CZ	3.82
LEU	448	CD2	PHE	458	CE1	3.87
LEU	448	CD2	PRO	535	CB	3.68
ASP	450	CB	PHE	446	CE2	3.52
LEU	452	CD2	PHE	458	CD2	3.79
GLN	453	NE2	SER	441	OG	3.6
TYR	454	CE2	GLY	440	CA	3.39
TYR	454	O	TYR	439	CE2	3.48
TYR	454	CE1	SER	441	N	3.67
TYR	454	CE2	PHE	446	CE2	3.8
TYR	454	OH	GLY	442	N	3.11
TYR	454	OH	LYS	444	O	2.65
GLU	457	OE2	SER	441	OG	2.77
GLU	457	C	TYR	439	OH	3.67
GLU	457	OE1	GLY	440	C	3.91
PHE	458	CD2	LEU	452	CD2	3.79
PHE	458	CE2	TRP	555	CB	3.81
PHE	458	CE1	LEU	448	CD2	3.87
PHE	458	CA	TYR	439	OH	3.23
PHE	458	CZ	ILE	559	CD1	3.94
ALA	459	CB	TRP	555	CH2	3.94
SER	467	C	ARG	432	NH2	3.14
SER	467	CB	TYR	436	OH	3.71
SER	467	O	ASP	562	OD1	3.34
PRO	468	C	ARG	432	NH2	3.26
PRO	468	N	ASP	562	OD1	3.93
VAL	469	N	ARG	432	NH2	3.42
ASP	470	CB	ARG	432	NH1	3.65

Chain B			Chain B'			Distance(Å)
HIS	534	NE2	GLN	431	O	3.93
HIS	534	CE1	ARG	432	CG	3.63
HIS	534	CD2	ARG	435	CB	3.19
HIS	534	O	TYR	436	OH	3.39
PRO	535	CB	LEU	448	CD2	3.68
PRO	535	CD	ARG	435	CG	3.46
PRO	535	CB	TYR	436	CE2	3.6
PRO	535	CG	TYR	439	CE1	3.89
PRO	537	CA	GLU	558	OE1	3.8
PRO	537	CB	TRP	555	NE1	2.92
ARG	538	NH1	ASP	562	OD2	3.45
ARG	538	N	GLU	558	OE1	2.97
HIS	539	CE1	ARG	554	NE	3.13
HIS	539	O	TRP	555	NE1	3.64
GLU	544	OE1	ARG	554	NH2	3.19
GLU	544	CD	CYS	551	SG	3.12
GLU	544	CA	VAL	547	CG1	3.85
GLU	544	OE2	SER	550	OG	3.91
VAL	547	CG1	GLU	544	CA	3.85
LEU	548	CD2	LEU	548	CD2	2.88
LEU	548	CD1	TRP	555	CH2	3.45
SER	550	OG	GLU	544	OE2	3.91
CYS	551	SG	GLU	544	CD	3.12
ARG	554	NH2	GLU	544	OE1	3.19
ARG	554	NE	HIS	539	CE1	3.13
TRP	555	CH2	ALA	459	CB	3.94
TRP	555	NE1	HIS	539	O	3.64
TRP	555	CB	PHE	458	CE2	3.81
TRP	555	NE1	PRO	537	CB	2.92
TRP	555	CH2	LEU	548	CD1	3.45
GLU	558	OE1	ARG	538	N	2.97
GLU	558	OE1	PRO	537	CA	3.8
ILE	559	CD1	PHE	458	CZ	3.94
ASP	562	OD2	ARG	538	NH1	3.45
ASP	562	OD1	PRO	468	N	3.93
ASP	562	OD1	SER	467	O	3.34

Tetramer interaction (Kink)

Chain B			Chain A'			Distance (Å)
GLN	381	OE1	GLN	431	NE2	3.04
ALA	382	O	GLN	431	CA	3.57
ALA	382	O	GLU	434	CB	3.62
LEU	383	CD1	ARG	435	NE	3.76
LEU	383	CD2	GLU	434	CG	3.37
LEU	383	O	GLN	431	CG	3.19
GLY	384	N	GLN	431	CB	3.78
GLU	387	OE2	GLY	440	O	3.29
GLU	387	OE2	SER	441	O	3.63
GLU	387	CD	ARG	435	NH2	3.31
GLU	387	OE2	ARG	445	NH2	2.57
HIS	390	CE1	GLY	442	O	3.41
HIS	390	CE1	SER	441	O	3.14
HIS	390	CD2	PRO	443	CG	3.62
GLN	431	CA	ALA	382	O	3.57
GLN	431	CB	GLY	384	N	3.78
GLN	431	CG	LEU	383	O	3.19
GLN	431	NE2	GLN	381	OE1	3.04
GLU	434	CG	LEU	383	CD2	3.37
GLU	434	CB	ALA	382	O	3.62
ARG	435	NE	LEU	383	CD1	3.76
ARG	435	NH2	GLU	387	CD	3.31
GLY	440	O	GLU	387	OE2	3.29
SER	441	O	GLU	387	OE2	3.63
SER	441	OG	GLY	601	C	3.9
SER	441	O	HIS	390	CE1	3.14
GLY	442	O	HIS	390	CE1	3.41
PRO	443	CG	HIS	390	CD2	3.62
PRO	443	CG	ARG	645	O	3.89
PRO	443	CD	ASN	646	OD1	3.68
ARG	445	NH2	GLU	387	OE2	2.57
GLY	601	C	SER	441	OG	3.9
ARG	645	O	PRO	443	CG	3.89
ASN	646	OD1	PRO	443	CD	3.68

b

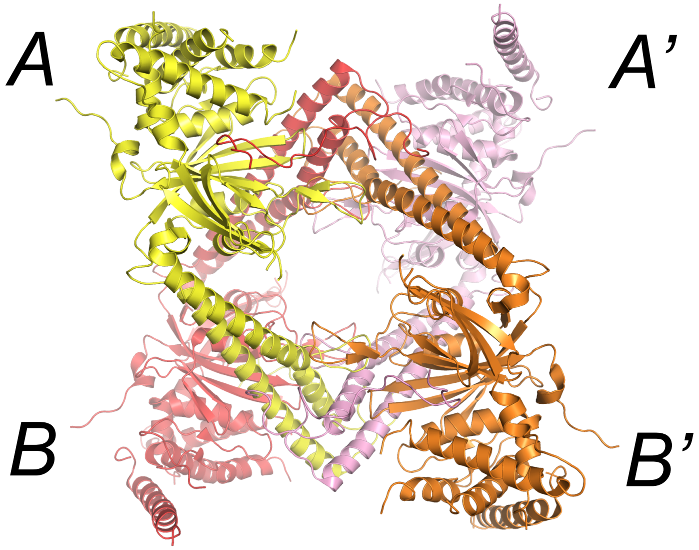

Supplementary Figure 5. Contact list of residues involved in the USP25 tetramer assembly, cutoff 4.0 Å.

(a) List of residues with interatomic distances less than 4.0 Å are shown. The interaction interface residues are generated by the online tool COCOMAPS. The interfaces between chains indicate tetramer, "*Il-loop*" and "*Kink*" (chains BA and BA', respectively) and dimer (chains BB') interactions. (b) Overall structure of the USP25 tetramer depicting its four chains labeled as A, A', B, and B'.

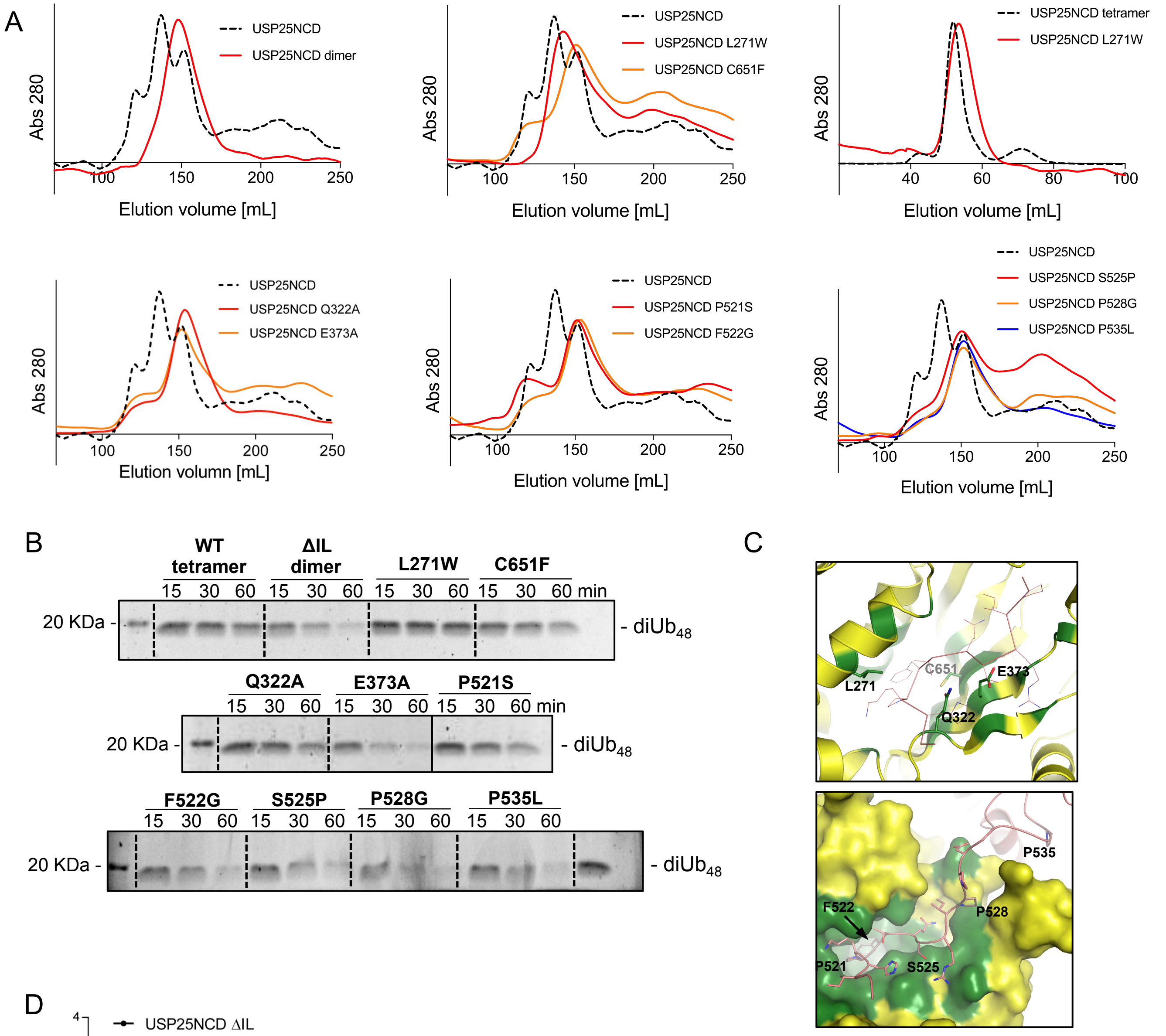

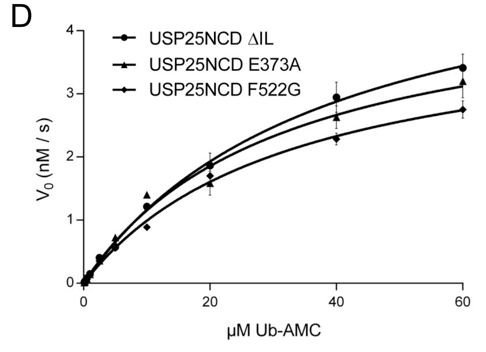

USP25NCD mutants	V_{max} (nM/s)	K_M (μM)	K_{cat} (s^{-1})	K_{cat}/K_M (M^{-1} $s^{-1}10^5$)
**E373A**	4.73 ± 0.37	31.01 ± 5.16	0.95 ± 0.07	0.305
**F522G**	4.23 ± 0.17	32.79 ± 2.76	0.85 ± 0.03	0.259
**ΔIL *dimer***	5.66 ± 0.31	38.68 ± 4.20	1.13 ± 0.06	0.293

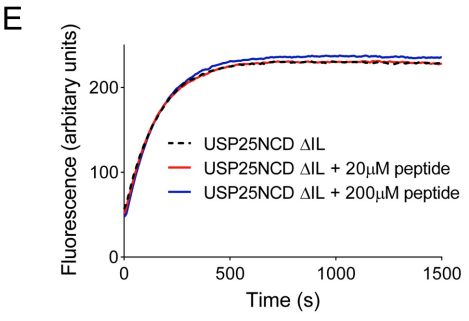

Supplementary Figure 6. Oligomerization and de-ubiquitination analysis of the USP25 point mutants of the IL-loop interface.

(A) Gel filtration profiles of the purifications of the different USP25 NCD point mutants of the interface between the IL-loop and the S1 ubiquitin-binding surface (see also Ub-AMC activities in figure 4e). (B) SDS-PAGE of the time-course reaction of the deubiquitinating activities on K_{48}-linked diUb substrate with the different USP25 NCD point mutants of the interface. (C) ***Above***, cartoon representation of the IL-loop interaction, indicating in stick representation the point mutant residues in the USP-like surface. ***Below***, surface and stick representation of the IL-loop interaction. Point mutant residues are labeled and shown in stick representation. (D) ***Left***, plot of the comparison of the "Michaelis-Menten" curves for USP25 NCD ΔIL dimer and point mutants F522G and E373A. Error bars represent the standard error for two independent experiments. ***Right***, table of the "Michaelis-Menten" parameters of USP25 point mutants F522G, E373A and ΔIL dimer. (E) Effect of the synthetic IL-loop peptide, at 20 and 200 μM, on the deubiquitinating activity of the USP25 NCD ΔIL "constitutive" dimer.

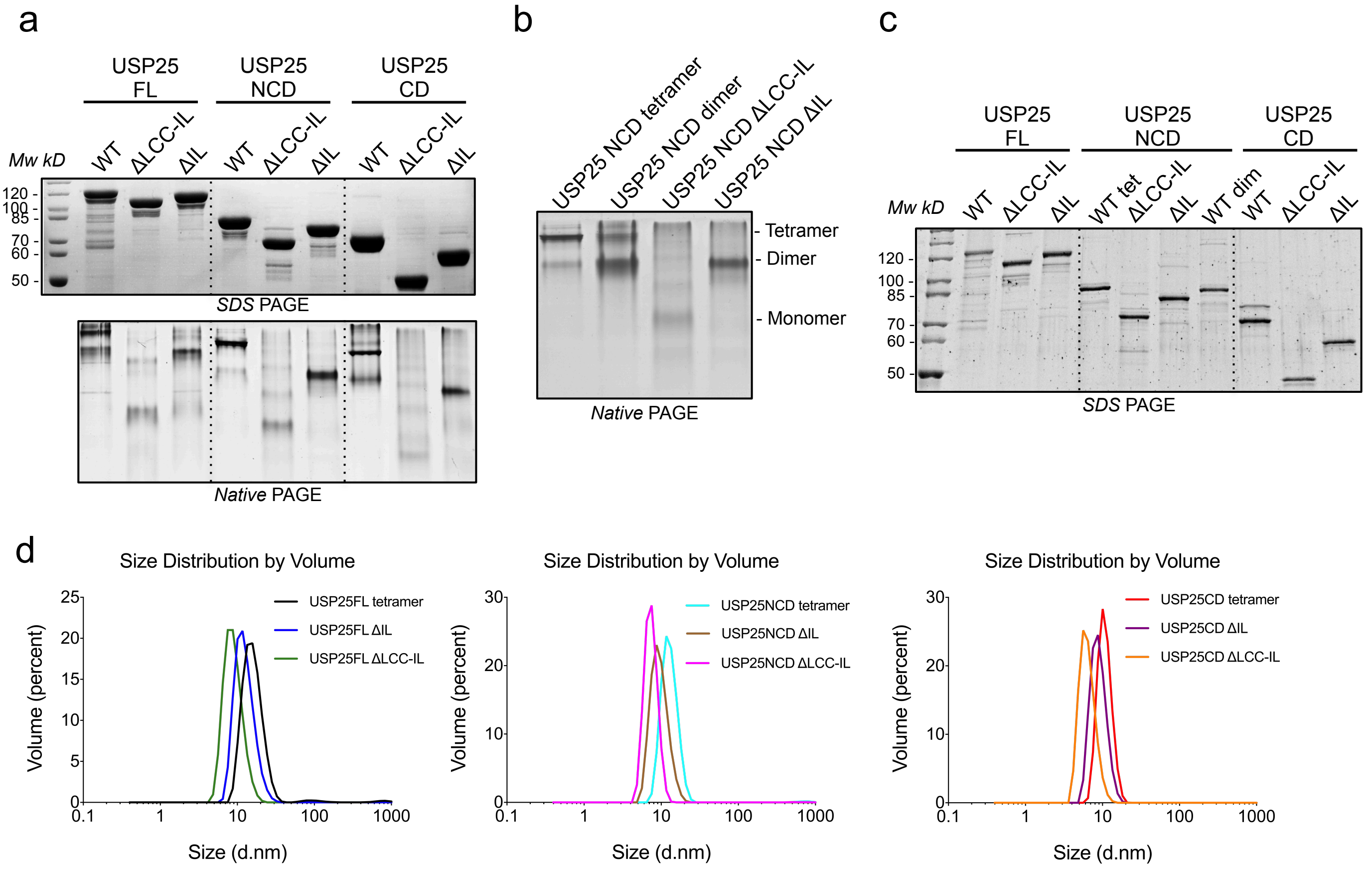

USP25	PDI	Size by volume (d.nm)	Mean Count Rate (kcps)	Estimated MW (kDa)	MW (kDa)
FL tetramer	1.000	16.10 ± 4.64	233.4	443	489
FL ΔIL	0.401	12.67 ± 3.80	108.0	253	230
FL ΔLCC-IL	0.471	9.03 ± 2.55	125.9	115	104
NCD tetramer	1.000	12.98 ± 3.03	196.8	268	321
NCD ΔIL	0.672	9.66 ± 2.53	257.0	134	146
NCD ΔLCC-IL	0.551	7.44 ± 1.43	160.7	72.9	62
CD tetramer	1.000	10.80 ± 2.21	225.6	174	266
CD ΔIL	0.911	8.99 ± 2.11	166.7	114	118
CD ΔLCC-IL	0.866	6.46 ± 1.55	79.5	52.5	48

Supplementary Figure 7. SDS/Native gels and dynamic light scattering of USP25 truncation constructs.

(a) SDS-PAGE (*above*) and native PAGE (below) of the different truncation constructs of USP25. (b) Native PAGE of the purified USP25 NCD tetramer and dimer, truncation monomer (USP25 NCD ΔLCC-IL), and truncation dimer (USP25 NCD ΔIL). Gels in A and B were stained with coomassie brilliant blue. (c) SDS-PAGE with the comparison of the amount of the USP25 truncation constructs used in the deubiquitinating activity assays in figure 5, stained with SYPRO-Ruby. (d) *Above*, size distribution by volume plots by dynamic light scattering of FL (right), NCD (middle) and CD (left) truncations of USP25. *Below*, table of the dynamic light scattering of the USP25 truncations.

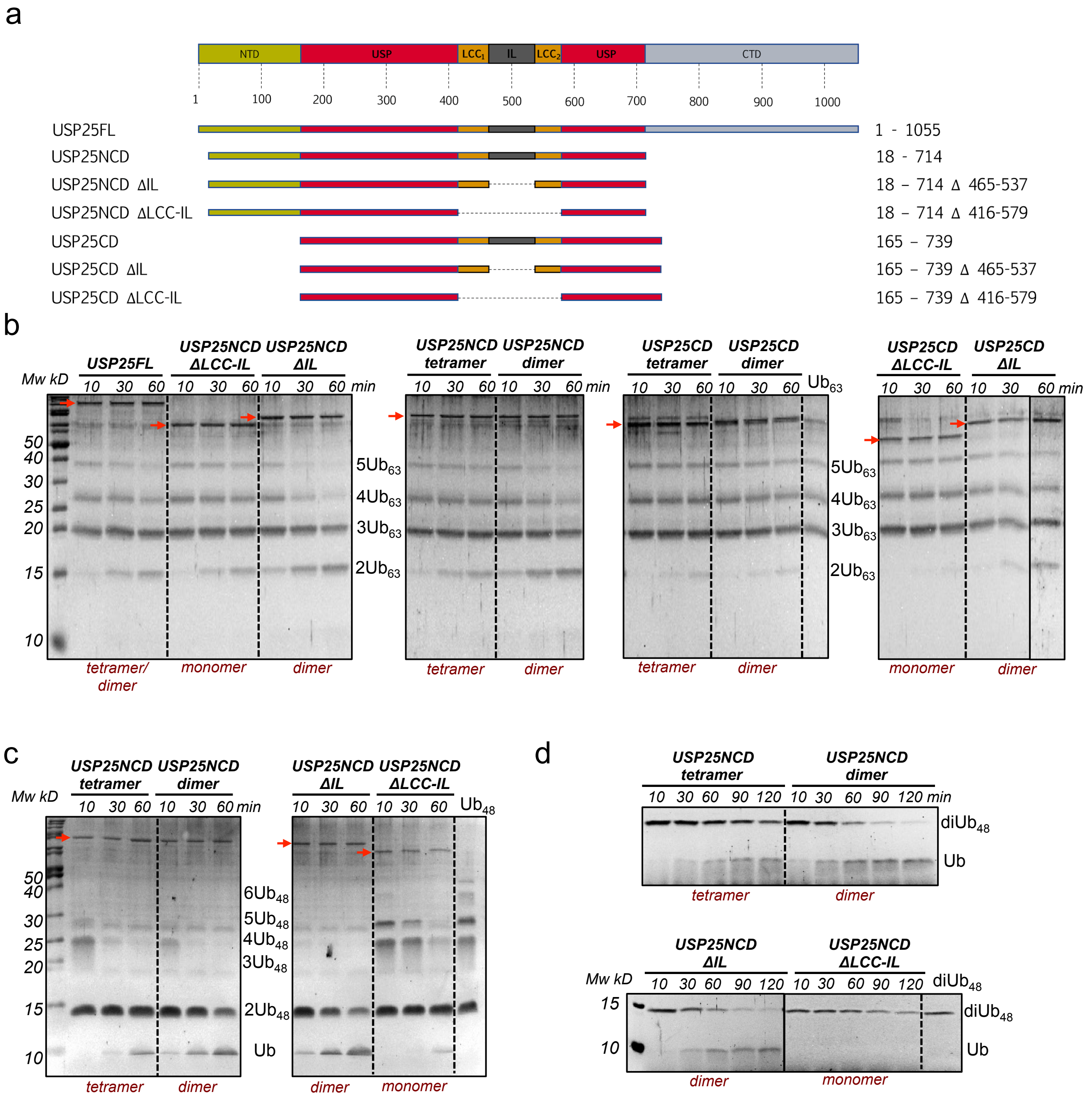

Supplementary Figure 8. Activity assays of USP25 truncation constructs with di-Ubiquitin and poly-Ubiquitin chains.

(a) Cartoon representation of the different truncation constructs of USP25. Left column indicate the names and the right column the residue range for each construct. (b) SDS-PAGE of the time course de-ubiquitinating assays with K_{63}-linked polyubiquitin substrate. Second column indicates the activities with purified tetramer and dimer USP25 fractions. Red arrows indicate USP25 truncation constructs. Reactions were stopped with SDS-loading buffer at indicated times. (c) SDS-PAGE of the time course de-ubiquitinating assays with K_{48}-linked poly-ubiquitin substrate. Left panels indicate the activities with purified tetramer and dimer USP25 fractions. Red arrows indicate USP25 truncation constructs. Reactions were stopped with SDS-loading buffer at indicated times. (d) SDS-PAGE of the time course de-ubiquitinating assays with K_{48}-linked diubiquitin substrate. Panels above indicate the activities with purified tetramer and dimer USP25 fractions.

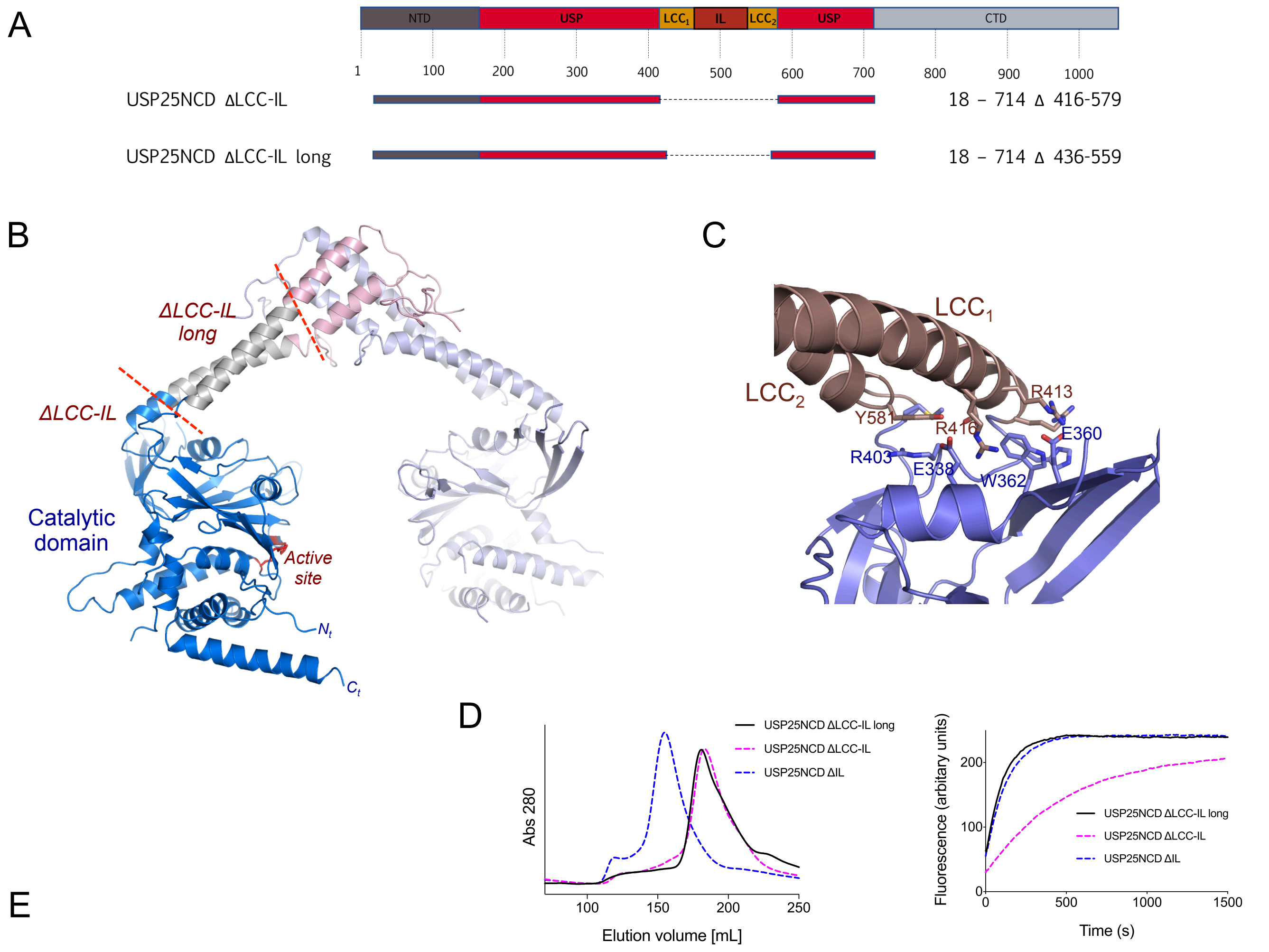

E

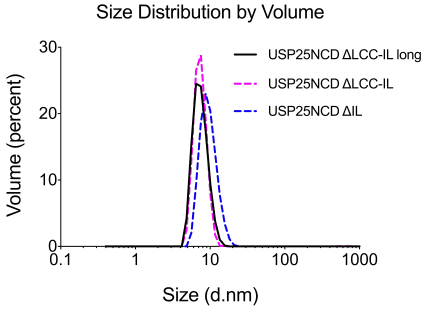

USP25 NCD	PDI	Size by volume (d.nm)	Mean Count Rate (kcps)	Est. MW (kDa)	MW (kDa)
ΔLCC-IL long	0.260	7.54 ± 1.76	49.2	75.2	67
ΔLCC-IL	0.551	7.44 ± 1.43	160.7	72.9	62

Supplementary Figure 9. Analysis of the two different USP25 monomer constructs. (A) Scheme representation with boundaries for the two different monomer constructs of USP25. (B) Cartoon representation of the two different USP25 monomers, indicating the truncation of the long and short monomer. (C) Zoom-up view of the contacts between the coiled-coil LCC domain and the USP25 catalytic domain. Major contacts are labeled and shown in stick representation. (D) ***Left***, gel filtration purification profiles comparing the dimer (USP25 *ΔIL)* and the two different monomers constructs (USP25 *ΔLCC-IL and USP25 ΔLCC-IL long).* ***Right***, plots of the de-ubiquitinating activities using Ub-AMC for the dimer (USP25 *ΔIL)* and the two different monomers constructs (USP25 *ΔLCC-IL and USP25 ΔLCC-IL long).* (E) Dynamic-light scattering analysis of the two different monomer constructs (USP25 *ΔLCC-IL and ΔLCC-IL long).*

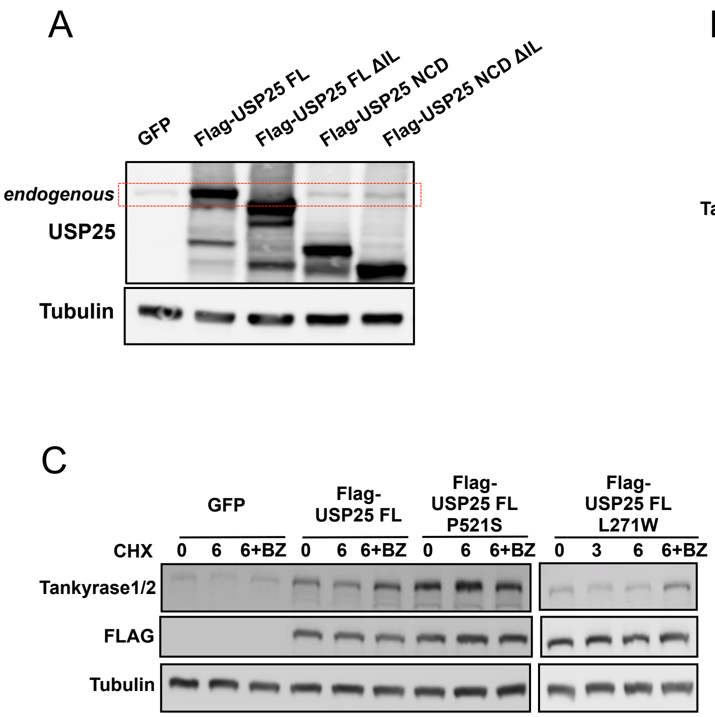

Supplementary Figure 10. Endogenous USP25 levels and immunoprecipitation analysis. (A) Flag-USP25FL, Flag-USP25FL ΔIL, Flag-USP25NCD and Flag-USP25NCD ΔIL were transfected in HEK293T cells and the levels of endogenous USP25 were analyzed by western-blot. GFP was transfected as a control. Dashed red rectangle indicates endogenous USP25. (B) Immunoprecipitation with anti-Flag resin of the HEK293T cells transfected with Flag-USP25FL, Flag-USP25FL ΔIL, Flag-USP25NCD and Flag-USP25NCD ΔIL plasmids. Endogenous tankyrases1/2 were analyzed by western-blot. (C) HEK293T cells were transfected with Flag-USP25FL, Flag-USP25FL P521S, Flag-USP25FL L271W and GFP and cells were treated with 100 μg/ml of cyclohexamide (CHX) and/or 0.5 μM of bortezomyb (BZ) and collected at 6 hours for western-blotting.

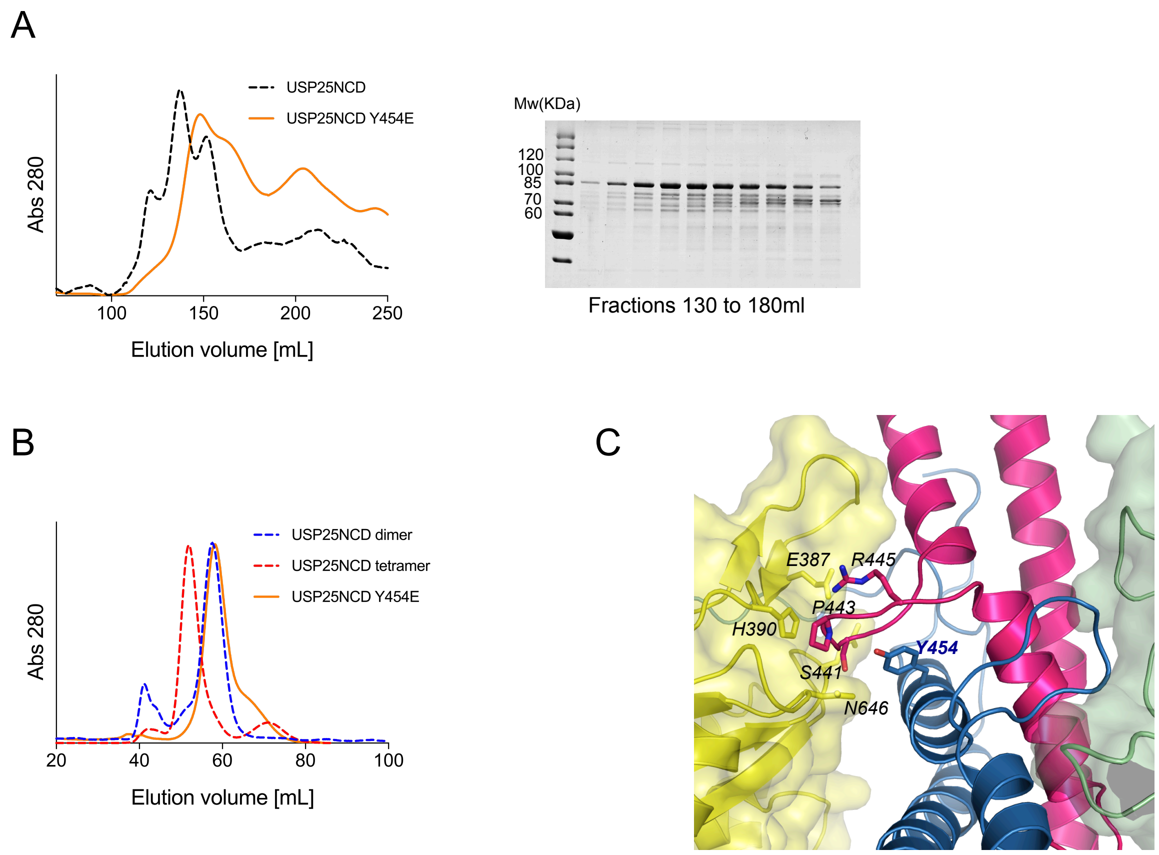

Supplementary Figure 11. USP25 Y454E point mutation analysis.

(A) ***Left***, gel filtration chromatography of the purification of wild-type USP25 and USP25 Y454E constructs using Superdex200 column. ***Right***, SDS-PAGE of the fractions (5mL/fraction) of the gel filtration peak of USP25NCD Y454E. Gel stained with coomassie blue staining. (B) Analytical gel filtration chromatography analysis of the USP25NCD tetramer, USP25NCD dimer and USP25NCD Y454E using Superdex16 column. (C) Zoomed up representation of the contact residues around Tyr454. Stick model of interface residues of the "*kink*" motif and the catalytic USP-like domain.

Figure 1 b

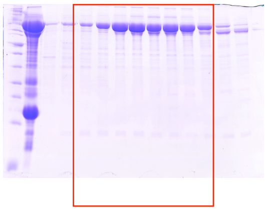

Figure 1 b

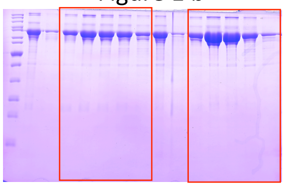

Figure 5 b

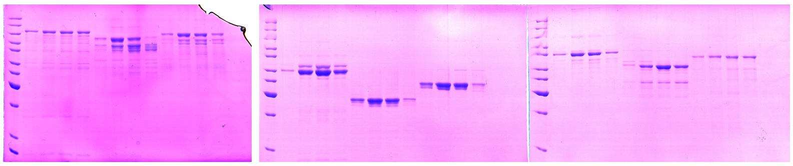

Supplementary Figure 2 A

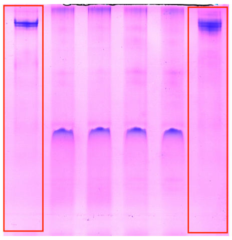

Supplementary Figure 2 C

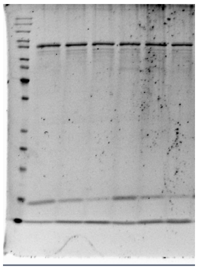

Supplementary Figure 2 E

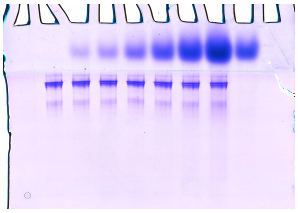

Supplementary Figure 2 F

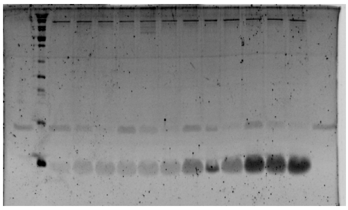

Supplementary Figure 6 B

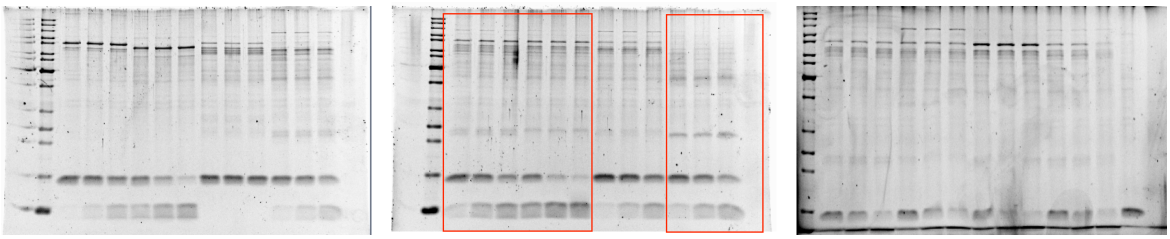

Supplementary Figure 7 A

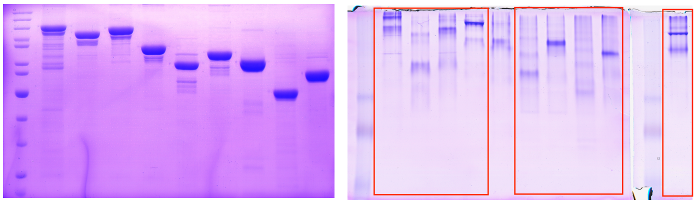

Figure 1 c and Supplementary Figure 7 B

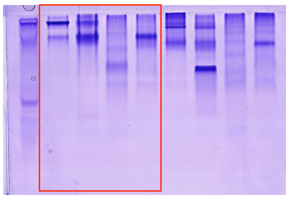

Supplementary Figure 7 C

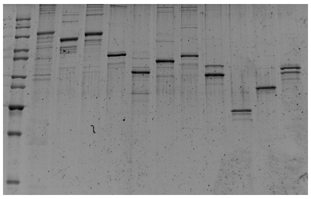

Supplementary Figure 8 B

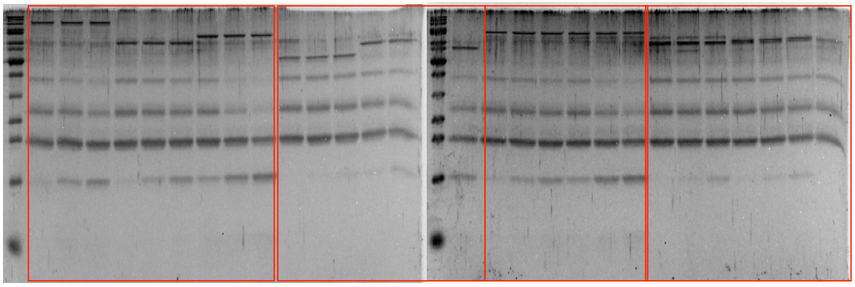

Supplementary Figure 8 C

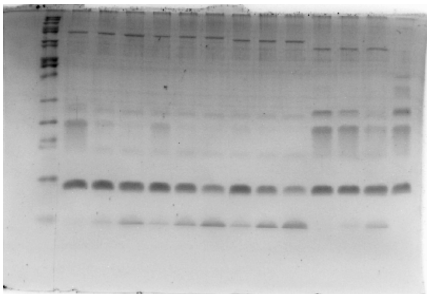

Supplementary Figure 8 D

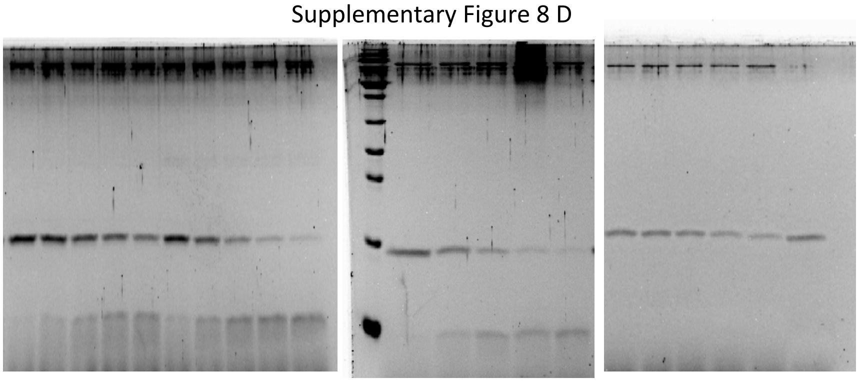

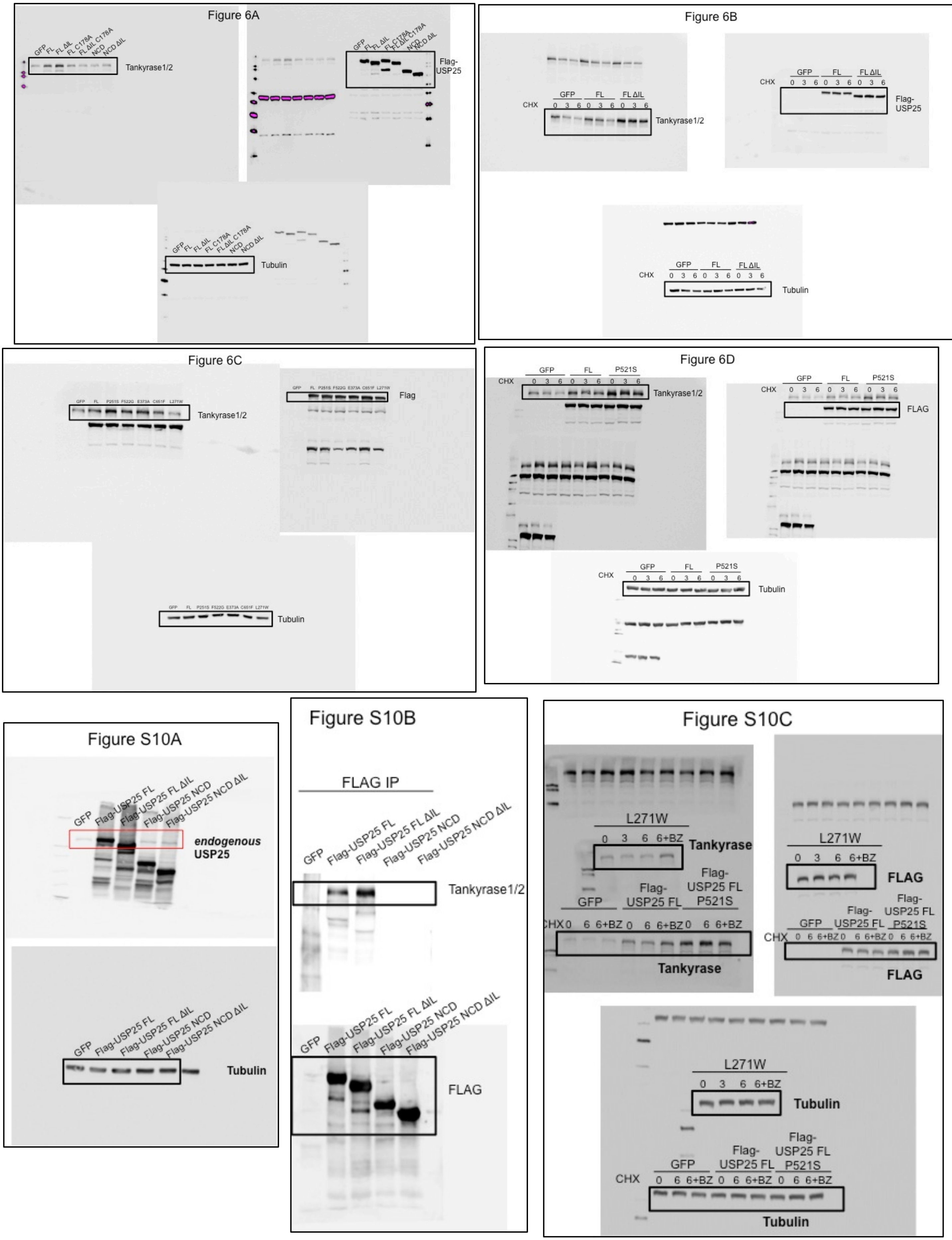

Supplementary Figure 12. Uncropped images of gels and blots.
Original images of PAGE gels and western blots (WB) displayed in the manuscript. For the WB the antibody utilized is depicted.

Supplementary Table 1. Primers used in this study

USP25FL_F	ATAGGATCCATGACCGTGGAGCAGAACGTG
USP25FL_R	ATAGCGGCCGCTTATCTTCCATCAGCAGGAGTTC
USP25CD_F	ATAGGATCCGACAAAGCTCCCGTTGGGCTAAAG
USP25NCD_F	ATAGGATCCCAGACGTTTTTGAATCAACTG
USP25NCD_R	ATAGCGGCCGCTTACTGCAAAGCTTTCTGG
USP25-739-stop_F	GCAGCAGGAGACCCATAATATCTAGAGCAGCCATC
USP25-739-stop_R	GATGGCTGCTCTAGATATTATGGGTCTCCTGCTGC
USP25del_464-538_R	GTGCCTACCCGATCCAACAGGTTTACTTGAGGCAA
USP25del_464-538_F	CTGTTGGATCGGGTAGGCACATAACGGAGGAAG
USP25del_415-580_R	GTACATACCCGATCCCTTAATTCTTGTTATTTCTC
USP25del_415-580_F	TTAAGGGATCGGGTATGTACTCTGACAAATCTATG
USP25_F522G_F	GTAATACACAAACCAGGTACTCAGTCCCGGATA
USP25_F522G_R	TATCCGGGACTGAGTACCTGGTTTGTGTATTAC
USP25_P535L_F	GATTTGCCCATGCATCTGGCACCAAGGCACATA
USP25_P535L_R	TATGTGCCTTGGTGCCAGATGCATGGGCAAATC
USP25_Y454F_F	TAGATGTTCTTCAGTTTGCATTGGAATTTGC
USP25_Y454F_R	GCAAATTCCAATGCAAACTGAAGAACATCTA
USP25_Y454E_F	GTAGATGTTCTTCAGGAGGCATTGGAATTTGCC
USP25_Y454E_R	GGCAAATTCCAATGCCTCCTGAAGAACATCTAC
USP25_L271W_F	GTTTACACACAAATTATGGGATTGGTTAGAAGAT
USP25_L271W_R	ATCTTCTAACCAATCCCATAATTTGTGTGTAAAC
USP25_C651F_F	ATGCCAGTGCATACTTTTTAATGTACATAAAT
USP25_C651F_R	ATTTATGTACATTAAAAAGTATGCACTGGCAT
USP25_E373A_F	CCTGTGTTAACATTTGCATTGTCAAGATTTGA
USP25_E373A_R	TCAAATCTTGACAATGCAAATGTTAACACAGG
USP25_Q322A_F	ACTGAAATGTTTGGTGCGTACCCACTTCAGGT
USP25_Q322A_R	ACCTGAAGTGGGTACGCACCAAACATTTCAGT
USP25_P521S_F	TCAGTAATACACAAAAGCTTTACTCAGTCCCGG
USP25_P521S_R	CCGGGACTGAGTAAAGCTTTTGTGTATTACTGA
USP25_S525P_F	CAAACCATTTACTCAGCCCCGGATACCTCCAG
USP25_S525P_R	CTGGAGGTATCCGGGGCTGAGTAAATGGTTTG
USP25_P528G_F	ACTCAGTCCCGGATAGGTCCAGATTTGCCCAT
USP25_P528G_R	ATGGGCAAATCTGGACCTATCCGGGACTGAGT
USP25_C178A_F	AATGTTGGCAATACTGCTTGGTTTAGTGCTGT
USP25_C178A_R	ACAGCACTAAACCAAGCAGTATTGCCAACATT
USP25del_436-559_R	TCTATACCCGATCCATATCTTTCTAGCCTTTGT
USP25del_436-559_F	GATATGGATCGGGTATAGAAAATGACACCAGAG
USP25seq1	GCTGGGCACTACTGGGCATAT
USP25seq2	GTGCCTAGAAGCTGCAATG
USP25seq3	GTTTGAAGAGGACACCTAC
Flag-USP25N-F	ATAGGATCCATGGATTACAAGGATGACGATGACAAGACCGTGGAGCAGAACGTG
