# Supplementary material for: A quaternary tetramer assembly inhibits the deubiquitinating activity of USP25
Source: Nat Commun. 2018 Nov 26;9:4973. doi: 10.1038/s41467-018-07510-5 (PMC6255862; doi:10.1038/s41467-018-07510-5)
Supplement: Supplementary file 3 — Reporting Summary [file 41467_2018_7510_MOESM3_ESM.pdf]

nature research

Corresponding author(s): DBPR NCOMMS-17-33044C

Reporting Summary

Nature Research wishes to improve the reproducibility of the work that we publish. This form provides structure for consistency and transparency in reporting. For further information on Nature Research policies, see Authors & Referees and the Editorial Policy Checklist.

Statistical parameters

When statistical analyses are reported, confirm that the following items are present in the relevant location (e.g. figure legend, table legend, main text, or Methods section).

n/a	Confirmed	
☐	☒	The exact sample size (n) for each experimental group/condition, given as a discrete number and unit of measurement
☒	☐	An indication of whether measurements were taken from distinct samples or whether the same sample was measured repeatedly
☐	☒	The statistical test(s) used AND whether they are one- or two-sided<br>*Only common tests should be described solely by name; describe more complex techniques in the Methods section.*
☒	☐	A description of all covariates tested
☒	☐	A description of any assumptions or corrections, such as tests of normality and adjustment for multiple comparisons
☒	☐	A full description of the statistics including central tendency (e.g. means) or other basic estimates (e.g. regression coefficient) AND variation (e.g. standard deviation) or associated estimates of uncertainty (e.g. confidence intervals)
☒	☐	For null hypothesis testing, the test statistic (e.g. F, t, r) with confidence intervals, effect sizes, degrees of freedom and P value noted<br>*Give P values as exact values whenever suitable.*
☒	☐	For Bayesian analysis, information on the choice of priors and Markov chain Monte Carlo settings
☒	☐	For hierarchical and complex designs, identification of the appropriate level for tests and full reporting of outcomes
☒	☐	Estimates of effect sizes (e.g. Cohen's d, Pearson's r), indicating how they were calculated
☐	☒	Clearly defined error bars<br>*State explicitly what error bars represent (e.g. SD, SE, CI)*

Our web collection on statistics for biologists may be useful.

Software and code

Policy information about availability of computer code

Data collection	N/A
Data analysis	N/A

For manuscripts utilizing custom algorithms or software that are central to the research but not yet described in published literature, software must be made available to editors/reviewers upon request. We strongly encourage code deposition in a community repository (e.g. GitHub). See the Nature Research guidelines for submitting code & software for further information.

Data

Policy information about availability of data

All manuscripts must include a data availability statement. This statement should provide the following information, where applicable:

- Accession codes, unique identifiers, or web links for publicly available datasets
- A list of figures that have associated raw data
- A description of any restrictions on data availability

Coordinates and reflection data files have been submitted to the Protein Data Bank with accession number 5O71. A reporting summary for this Article is available as a Supplementary Information file. Other data are available from the corresponding author upon reasonable request.

Field-specific reporting

Please select the best fit for your research. If you are not sure, read the appropriate sections before making your selection.

- [x] Life sciences
- [ ] Behavioural & social sciences
- [ ] Ecological, evolutionary & environmental sciences

For a reference copy of the document with all sections, see nature.com/authors/policies/ReportingSummary-flat.pdf

Life sciences study design

All studies must disclose on these points even when the disclosure is negative.

Sample size	Kinetics analysis of truncation mutants and point mutants was conducted in duplicate. Tankyrase levels. Data are represented as mean ± SD of three or more independent experiments. Statistical tests were performed using LAS4000 and ImageJ 1.49v softwares. Comparison between samples was evaluated by independent sample t test, and results were considered statistically significant when p-val<0.05.
Data exclusions	Data not excluded
Replication	Data represent 3 or more replicates
Randomization	Not relevant for the study, samples are not random.
Blinding	Not relevant for the study, samples not blinded.

Reporting for specific materials, systems and methods

Materials & experimental systems

n/a	Involved in the study	
[x]	[ ]	Unique biological materials
[ ]	[x]	Antibodies
[ ]	[x]	Eukaryotic cell lines
[x]	[ ]	Palaeontology
[x]	[ ]	Animals and other organisms
[x]	[ ]	Human research participants

Methods

n/a	Involved in the study	
[x]	[ ]	ChIP-seq
[x]	[ ]	Flow cytometry
[x]	[ ]	MRI-based neuroimaging

Antibodies

Antibodies used	Anti-TNKS1/2 (1:1000 dilutions; Santa Cruz Biotechnology; sc-8337); anti-[illegible]-catenin (1:1000 dilution; Cell signaling Technology, no. 9562), anti-Flag (1:1000 dilutions; Sigma-Aldrich, F7425) and anti-Tubulin (1:5000 dilutions; Sigma, T5168).
Validation	Commercial antibodies validated

Eukaryotic cell lines

Policy information about cell lines

Cell line source(s)	human embryonic kidney 293T cell line (HEK293T) (CRL-1573; ATCC)
Authentication	Purchased from ATCC (CRL-1573; ATCC)
Mycoplasma contamination	Tested and not contaminated
Commonly misidentified lines (See ICLAC register)	N/A
